# Sugar-sweetened beverage taxes: Lessons to date and the future of taxation

**DOI:** 10.1371/journal.pmed.1003412

**Published:** 2021-01-07

**Authors:** Barry M. Popkin, Shu Wen Ng

**Affiliations:** Department of Nutrition, Gillings School of Global Public Health and the Carolina Population Center, The University of North Carolina Chapel Hill, North Carolina, United States of America

To date, across the globe, over 45 countries, cities, and regions have instituted sugar-sweetened beverage (SSB) taxes. Fig 1 is a map of the world highlighting the diversity of countries where SSB taxes now exist and options in tax formats [1].

**Fig 1:**
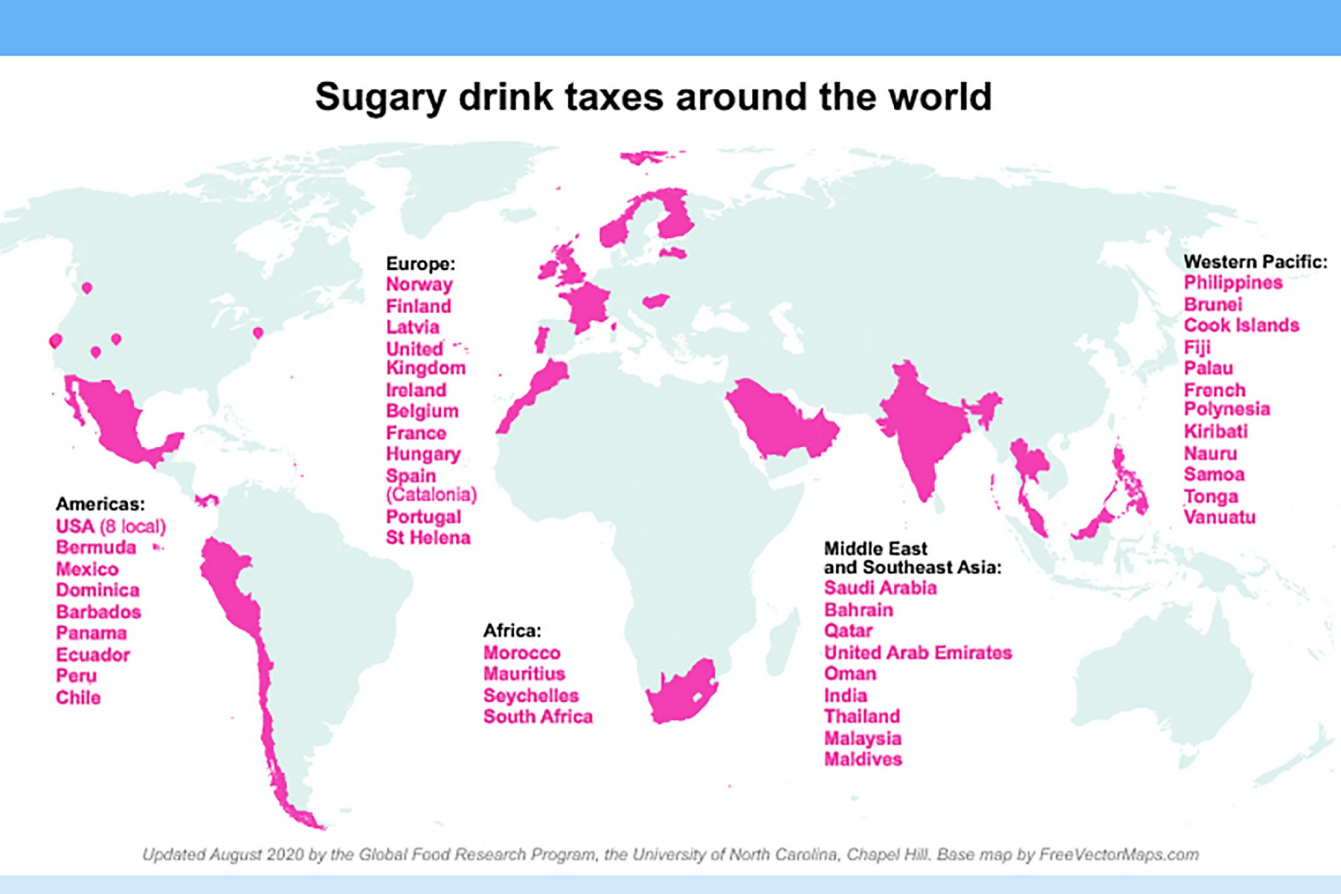
Global map of countries with SSB taxes. SSB, sugar-sweetened beverage.

## Why tax SSBs?

We see at least 2 major health-related reasons to focus on SSBs. First, excess sugar consumption is a major cause of obesity and the increasing risk of type 2 diabetes, hypertension, liver and kidney damage, heart disease, and some cancers [2–4]. Second, high-caloric beverages offer little caloric compensation, so reducing their consumption lowers obesity risk [5,6]. Furthermore, we are beginning to understand the potential impacts of these beverages on stunting as well as obesity [7–11]. A less emphasized reason that deserves greater attention as we consider the links between our diets and planetary health is the environmental costs related to the production of sugary drinks, particularly in water use and carbon emissions. Estimates of total water lifecycle costs to produce a half-liter (or 17-ounce) regular soft drink range from 168 to 309 liters, depending on the sugar source and farm technology [12–14]. Finally, from a practical standpoint, SSB taxes have gained momentum because of their relative ease of implementation compared to other food/nutrition policy options. Taxes collected from manufacturers, bottlers, and distributors can often be built into existing taxation frameworks and collection systems, and these health taxes are a potential source of revenue.

## What have we learned from major evaluations to date?

Three recent papers published in *PLOS Medicine* highlight the potentials of different methods of discouraging SSB consumption among the public and encouraging reformulations by the beverage industry. In the United Kingdom, the multitiered Sugar Drinks Industry Levy based on sugar content has prompted remarkable reformulations and shifts in purchases with new low-calorie beverages emerging [15]. In Portugal, earlier findings suggest both sugar reduction in beverage formulations and reduced sales [16], and the latest paper by Goiana-da-Silva and colleagues simulates its implication for lowering new cases of obesity in children, adolescents, and adults [17]. Likewise, reformulations are an important driver of change in response to Chile’s integrated food labeling, marketing, and school food regulations [18]. Taillie and colleagues’ new study found a 23.7% reduction overall in the volume of SSBs purchased and a 27.5% decline in calories consumed per capita per day [19]. While reductions in SSB purchases and modeled improvements in obesity outcomes address the objectives of these policies, the implications of reformulations are unclear and need monitoring.

Overall, price changes are heterogeneous depending on the baseline levels of consumption, market shares of beverage brands given the geographic coverage of the tax, and reflect strategic behaviors by beverage companies and retailers [20,21]. Consequently, changes in consumption are also heterogeneous, particularly across income levels, age groups, and baseline beverage levels [21–23]. Nonetheless, a meta-analysis shows that the average consumer will lower his/her SSB purchases by 10% if SSB prices rise 10% (price elasticity of demand of −1) [24]. Health implications (e.g., weight change, flattening of diabetes prevalence rates, or reductions in obesity incidence) take years to emerge at the population level, so researchers have used the available results to estimate longer-term health and economic implications, such as in the recent analysis by Goiana-da-Silva and colleagues [17].

The findings to date suggest that future SSB tax designs need to consider the baseline levels of consumption of various beverages stratified by income and the price elasticities of demand that can guide the scope of the products covered. Second, the tax structure should be aligned with the primary objectives. If the goal is reduction of sugar consumption, then one based on sugar density is more likely to achieve the goal, as the Portugal and UK results suggest. If the goal is revenue generation, then a volume-based specific tax across a broad scope of beverages may result in greater revenue given a weaker incentive to reformulate. Meanwhile, studies have shown that ad valorem taxes on SSBs are less likely to be fully pass through onto prices compared to specific taxes in the form of sales taxes (rather than excise taxes) [25]. Finally, the geographic coverage of the tax jurisdiction has implications for ease of cross-border shopping and highlights the need for national- or province/state-level taxes over local taxes.

A critical concern from the health perspective is the reductions in sugar and caloric intakes through reduced SSB consumption. While these taxes specifically affect high consumers [22,26], evaluations to date suggest that the reductions affected by SSB taxes translate to 5 to 22 kilocalories (kcals) per capita per day. These levels of reductions, even if sustained, are insufficient to meaningfully impact the broad swath of health outcomes in a timely manner, although research shows that the 10- to 20-year time horizon will produce important results [27,28]. One way to address this is to raise the current tax rates that are in the 5% to 20% range. A few Middle Eastern countries (e.g., Saudi Arabia, Qatar, and the United Arab Emirates) have instituted 50% to 100% excise taxes on subsets of SSBs [29], and Bermuda has implemented a 75% import tax on sugar, SSBs, and candies [30]. We can learn from tax levels for tobacco (another product with no health benefits and many costs) where the taxation rates range from 100% to 1,000% [31,32].

## Potential unknown consequences

Evidence to date demonstrates that sugar reduction policies will and have resulted in the introduction of beverages with both sugar and nonnutritive sweeteners (NNSs) [15,16,33–36].

NNS consumption is growing in high-income countries, but it is less clear what will happen in low- and middle-income countries, where consumption of diet beverages is minimal [36]. The Mexican and Berkeley evaluations found a shift toward water [37,38]. The few studies in low-income countries have found a small movement toward NNS-sweetened beverages [39], but that might change with large tax rates on SSBs or ultraprocessed foods.

So why the concern around NNSs? One fear is the impact on sweetness preference and habituation among children. Among adults, we see different outcomes in widely conflicting human studies looking at gut health, brain response, and heart health [40–43]. At present, no global consensus on the longer-term health implications of prolonged and/or larger doses of NNS intake exists. The dearth of information on their use (the types and amounts) in our food supply means that it is challenging to study these questions.

A few Latin American countries are exploring front of package labeling to inform consumers if products contain NNSs. Additional information on the amounts of the various NNSs will allow monitoring of our exposure to these additives and population-based observational studies on health outcomes of the types and amounts of NNSs in diets. Currently, Chile is the only country, to our knowledge, that requires the amount of each type of NNS on nutrition labels.

Of course, the food industry constantly undertakes research and development, and the scientific community’s understanding of how the various combinations of foods, ingredients, and chemicals we are exposed to affects our health over time is still growing. Any new regulations targeting current attributes of concern will meet subsequent introductions of new ingredients and products to avoid or minimize such regulations. Researchers and regulatory agencies must be vigilant and thoughtful in establishing mechanisms with which to periodically assess and improve these regulations to ensure that they evolve with the food landscape to best protect people’s health.

## Conclusions

Taxation of SSBs is an important start to using fiscal policy to correct the large human and planetary costs of the modern food supply chain and promote improved diet and eventually health [44]. SSB taxes to date have varied in design, and continued assessments can allow us to better understand how to improve them to sharpen their effects. To date, tax rates are often too low, and the net impact, while important for public health, needs to be increased significantly. Increasing SSB taxation levels or expanding the tax base to include unhealthy ultraprocessed foods and beverages offer options. Additionally, the tax revenues should be directed toward human capital investments, particularly those targeting lower-income individuals or households, to address equity concerns and strengthen public support. Regardless, careful monitoring of industry responses to taxes is important due to industry investments in new food technologies with unknown, longer-term implications on human and planetary health.

## Abbreviations

kcal: kilocalorie
NNS: nonnutritive sweetener
SSB: sugar-sweetened beverage
